# Cerebral Hemodynamics Measured by Wearable Near-Infrared Spectroscopy During Bedside Mobilization in a Patient With Chronic Heart Failure Hospitalized for Acute Exacerbation

**DOI:** 10.7759/cureus.78489

**Published:** 2025-02-04

**Authors:** Yu Takada, Naoyuki Hashimoto, Masafumi Kubota, Atsuhiro Tsubaki

**Affiliations:** 1 Department of Rehabilitation, Kanazawa University Hospital, Kanazawa, JPN; 2 Department of Physical Therapy, Faculty of Health Sciences, Institute of Medical, Pharmaceutical, and Health Sciences, Kanazawa University, Kanazawa, JPN; 3 Institute for Human Movement and Medical Sciences, Niigata University of Health and Welfare, Niigata, JPN

**Keywords:** bedside mobilization, cerebral hemodynamics, heart failure, near-infrared spectroscopy, wearable medical device, wearable technology

## Abstract

This report aimed to investigate the relationship between cerebral hemodynamics and orthostatic hypotension (OH) in a patient with acute exacerbation of chronic heart failure (HF) by measuring oxyhemoglobin (O_2_Hb) and regional cerebral oxygen saturation (rSO_2_) using the wearable near-infrared spectroscopy (NIRS) device for risk management. A 61-year-old man was diagnosed with acute exacerbation of chronic HF. The patient was admitted to the hospital for the first time on day 0, and the first NIRS was performed on day 9. He was discharged on day 30 but was admitted for the second time on day 86, and the second NIRS was performed on day 100.

Although HF symptoms, including weight gain, cardiomegaly, and pleural effusion, present at both admissions had improved at the time of NIRS measurements, there was a difference between the first and second admissions in diuresis, progression of diuresis-related weight loss, and OH symptoms. Specifically, weight loss progressed more rapidly during the first admission, with lower body weight at the time of the first NIRS measurement. Near-infrared spectroscopy assessments were conducted in the following sequence: supine, 30° head-up, sitting, standing, sitting, and supine again. During the first measurement, blood pressure decreased in the sitting and standing positions, heart rate increased only in the standing position, and the patient reported dizziness in both positions. At the second admission, blood pressure and heart rate fluctuated less, and there were no subjective symptoms. Oxyhemoglobin and rSO_2_ were lowest in the standing position in both measurements. However, compared with the second measurement, the first measurement showed greater variability in O_2_Hb and rSO_2_ and lower values in all positions except supine. These findings suggest that NIRS data may reflect changes in blood pressure, OH multiple variants, OH severity, and cerebral autoregulation. Additionally, they may be influenced by various factors, including differences in the progression of weight loss between the two hospitalizations.

Therefore, this study demonstrates the potential of wearable NIRS technology to transform patient care by providing real-time, actionable insights into cerebral hemodynamics. However, further research is required to confirm the generalizability of these findings.

## Introduction

Orthostatic hypotension (OH) is a common complication in patients with heart failure (HF) [1], closely associated with the risk of HF [2], and linked to an increased risk of HF events, particularly in patients with type 2 diabetes and hypertension [3]. Conversely, HF itself is associated with an elevated risk of OH, which can significantly impact symptoms and activity levels [4]. In severe cases, OH may lead to syncope, necessitating effective risk management strategies [5]. Harms et al. [6] have reviewed such strategies, emphasizing the utility of noninvasive methods to assess cerebral circulatory responses to orthostatic stress [7]. Their findings highlight the importance of clinical assessments to differentiate various OH subtypes, including initial OH, delayed blood pressure (BP) recovery, and sustained OH, across all age groups [6]. Evidence from healthy adults indicates that the duration of hypotension and cerebral hypoperfusion, rather than the magnitude of BP changes, is a critical determinant of syncope [8]. This highlights the clinical importance of evaluating factors beyond BP, with a specific focus on cerebral circulation. Wearable near-infrared spectroscopy (NIRS) devices offer a promising solution, enabling real-time, continuous monitoring of cerebral hemodynamics and oxygenation [9,10]. Their portability, ease of setup, and minimal movement restrictions allow measurements to be conducted during exercise therapy, bedside mobilization, and daily life activities, providing a practical advantage in evaluating OH.

Traditional BP measurements, which are often intermittent and cumbersome, may fail to capture the dynamic physiological changes associated with OH. By incorporating wearable NIRS technology into routine practice, it is possible to bridge the gap between episodic measurements, clinical findings, and dynamic physiological changes. This integration could enhance the accuracy of OH detection and improve risk management.

While wearable devices have gained attention in various medical fields, their potential for cardiac rehabilitation is increasingly recognized [11]. Considering the strong association between OH and HF, wearable NIRS technology represents an innovative tool for managing OH in the complex HF population. However, data on cerebral hemodynamic responses to postural changes in HF patients remain scarce, particularly during bedside mobilization and in cases of acute exacerbation of chronic HF [12].

In this single-case report, we investigated cerebral hemodynamics using a wearable NIRS device in a patient with acute exacerbation of chronic HF during bedside mobilization. We aimed to elucidate the relationship between cerebral hemodynamics and OH during mobilization in a patient with acute HF, contributing to the understanding of its clinical implications and potential management strategies.

This article was previously presented as a meeting abstract at the 2023 International Society on Oxygen Transport to Tissue (ISOTT) in Tokyo on September 30, 2023.

## Case presentation

Patient information

A Japanese man (height 177 cm) in his early 60s was admitted to the hospital for an acute exacerbation of chronic HF. His HF was due to systolic dysfunction with underlying ischemic heart disease (IHD) and the dilated phase of hypertrophic cardiomyopathy (D-HCM). His medical history included hypertension, type 2 diabetes mellitus, dyslipidemia, and chronic kidney disease. He was being treated for hypertension, diabetes mellitus, and dyslipidemia at a hospital near his home. Before admission, he lived alone and was independent.

**Figure 1:**
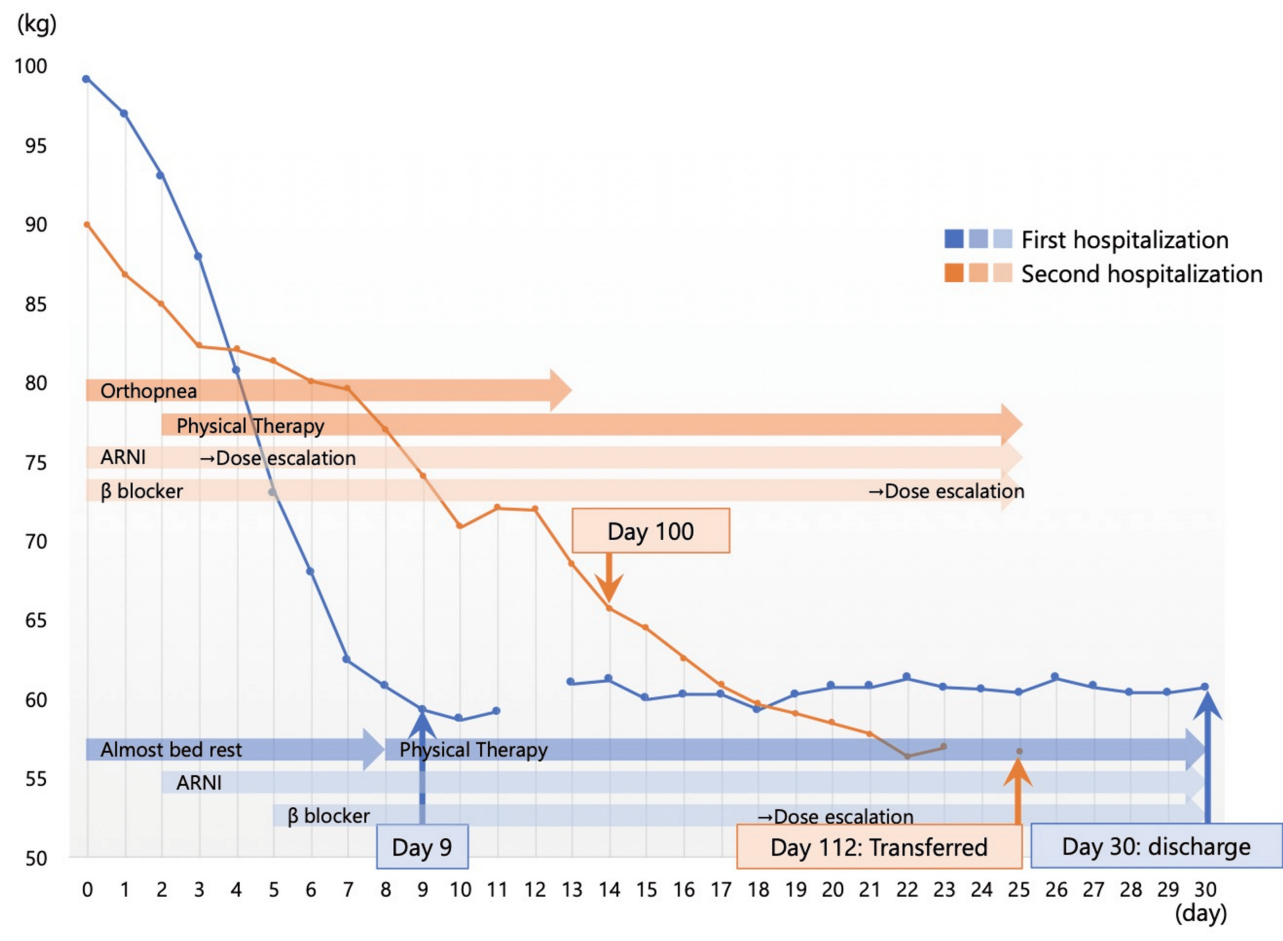
Timeline during first and second hospitalization Blue markers and lines: first hospitalization; orange markers and lines: second hospitalization ARNI: angiotensin receptor-neprilysin inhibitor; β: beta

Nine days before his initial admission, he became aware of leg edema and dyspnea, which progressed to orthopnea five days before admission. He was diagnosed with exacerbation of chronic HF on day 0 and was admitted to the hospital for the first time. The patient was classified as New York Heart Association (NYHA) functional classification IV. Chest radiography revealed a cardiothoracic ratio (CTR) of 62%, significant cardiomegaly, bilateral pleural effusion, and vascular congestion. Laboratory tests showed an N-terminal pro-brain natriuretic peptide (NT-proBNP) level of 24,014 pg/mL, blood urea nitrogen (BUN) of 24 mg/dL, creatinine (Cr) of 1.19 mg/dL, estimated glomerular filtration rate (eGFR) of 49.52 mL/min/1.73m², hemoglobin (Hb) of 9.6 g/dL, hematocrit (Ht) of 31.1%, albumin (Alb) of 2.3 g/dL, C-reactive protein (CRP) of 0.59 mg/dL, and pH of 7.451. Vital signs included a BP of 153/105 mmHg, a heart rate (HR) of 112 beats/min, and peripheral oxygen saturation (SpO₂) of 95% on room air. The patient weighed approximately 99 kg and exhibited peripheral coldness and lower leg edema. Nitroglycerin and furosemide were administered, and noninvasive positive pressure ventilation (NPPV) was initiated. On day 2, NPPV was discontinued and replaced with 2 L of nasal oxygen. The patient remained bedbound until day 6. On day 7, oxygen therapy was discontinued, and bedside sitting and wheelchair transfers were initiated. On day 8, a detailed echocardiographic examination revealed a left ventricular ejection fraction (LVEF) of 27% (Table 1). Physical therapy commenced on the same day, with an NYHA functional classification of III. Vital signs were BP of 95/63 mmHg, HR of 87 beats/min, and SpO₂ of 98% on room air. Peripheral coldness resolved, although lower leg edema persisted. Functional assessments showed a Clinical Frailty Scale (CFS) score of seven, Manual Muscle Testing (MMT) grades of three to four for major limb muscles, a Short Physical Performance Battery (SPPB) score of four (balance test: three points; gait test: one point; chair stand test: 0 points), and a Barthel Index (BI) score of 35 for activities of daily living (ADL). Diuresis progressed rapidly, and by day 9, the patient’s weight had decreased to 59.3 kg when the first NIRS measurement was performed. The patient was treated with an angiotensin receptor-neprilysin inhibitor (ARNI), a beta-blocker, and a selective sodium-glucose cotransporter-2 (SGLT2) inhibitor while undergoing the staged bedside mobilization program. By day 12, he could walk approximately 100 meters without resting using an IV pole for support. On day 16, resistance training and aerobic exercise on a bicycle ergometer began in the physical therapy room, and group rehabilitation commenced on day 17. No further HF exacerbations occurred, and the patient regained independence in ADL. Coronary angiography revealed 75% stenosis in the left anterior descending (LAD) artery, and on day 25, percutaneous coronary intervention was performed for the LAD. Although myocardial biopsy revealed no specific findings, comprehensive assessments, including MRI, led to a final diagnosis of IHD and D-HCM. The patient’s CTR improved to 45%, and he weighed 60.7 kg at discharge to home on day 30. The course of the first hospitalization is shown in Figure 1 (blue).

**Table 1:** Echocardiographic parameters LA: left atrial; LV: left ventricular; LVEF: left ventricular ejection fraction; RV: right ventricular

Date examined	Day 8	Day 107
LA diameter (mm)	35.3	42.7
LV end-diastolic diameter (mm)	57	61
LV end-systolic diameter (mm)	50	51
LVEF (%)	27	39
RV systolic pressure (mmHg)	18.3	24.6

However, about 1.5 months later, he developed leg edema and dyspnea, and he gradually gained weight. The dyspnea continued to worsen, and the patient was admitted to the hospital for the second time on day 86. The patient was classified as NYHA functional classification IV. Chest radiography revealed a CTR of 60%, significant cardiomegaly, bilateral pleural effusion, and vascular congestion. Laboratory tests showed an NT-proBNP level of 33,439 pg/mL, BUN of 25 mg/dL, Cr of 1.03 mg/dL, eGFR of 52.67 mL/min/1.73 m², Hb of 8.2 g/dL, Ht of 21.2%, Alb of 1.9 g/dL, CRP of 0.46 mg/dL, and pH of 7.412. Vital signs included a BP of 186/116 mmHg, an HR of 95 beats/min, and SpO₂ of 94% on 3 L via nasal cannula. The patient weighed approximately 90 kg and exhibited significant lower leg edema without peripheral coldness. Nitroglycerin and furosemide were administered, with oxygen therapy maintained at 3 L via nasal cannula. On day 87, the patient’s Hb level decreased to 6.6 g/dL, prompting a blood transfusion, which improved Hb to 8.4 g/dL by day 91. Wheelchair transfers began on day 87, followed by the initiation of physical therapy and ambulation to the room toilet on day 88. At this time, the patient was classified as NYHA functional classification III. Vital signs included a BP of 158/100 mmHg, an HR of 86 beats/min, and SpO₂ of 98% on 2 L via nasal cannula. Physical examination revealed persistent lower leg edema without peripheral coldness. Functional assessments showed a CFS score of six, MMT grades of three to four for major limb muscles, an SPPB score of four (balance test: three points; gait test: one point; chair stand test: 0 points), and a BI score of 50 for ADL. Diuresis progressed gradually compared to the first hospitalization. From days 88 to 95, intravenous atrial natriuretic polypeptide was administered. By day 100, during the second NIRS measurement, the patient’s weight had decreased to 65.7 kg. Medication doses, including an ARNI, a beta-blocker, and an SGLT2 inhibitor, were adjusted, and the staged mobilization was implemented. On day 99, the patient walked approximately 50 m without rest using an IV pole and began resistance training and aerobic exercise on a bicycle ergometer in the physical therapy room. Group rehabilitation sessions commenced on day 102. No further HF exacerbations occurred. By day 107, the patient’s LVEF had improved to 39%, and the SPPB score had increased to seven (balance test: four points; gait test: three points; chair stand test: 0 points). The patient achieved ADL independence. The patient’s CTR improved to 46%, and body weight decreased to 57.2 kg. To further enhance physical function, disease management, and relapse prevention, the patient was transferred to a nearby hospital on day 112. The course of the second hospitalization is shown in Figure 1 (orange).

Measurement and analysis protocol

The brain activity monitor, Hb133 (Astem Co., Ltd., Kawasaki, Japan), was used to measure NIRS in the right and left frontal regions. The measurement parameters were oxyhemoglobin (O_2_Hb), deoxyhemoglobin (HHb), total hemoglobin (THb), and regional cerebral oxygen saturation (rSO_2_). In the present study, we used O_2_Hb and rSO_2_ as parameters to analyze, referring to previous studies [13,14] that reported their potential use as markers of cerebral hemodynamics.

The Hb133 was a two-channel device with an optical mean power of less than 1 mW, peak wavelengths of 770 ± 10 nm and 830 ± 10 nm, and a sampling frequency of 10 Hz. Measurements were taken on day 9 during the first hospitalization and on day 100 during the second hospitalization. On both days, the patient changed postures in the following order: supine, 30° head-up (HUP), sitting, standing, sitting, and supine. Each posture was maintained so that NIRS data were obtained for at least 1.5 minutes for each posture. However, in the initial sitting and standing postures, subjects moved to the next posture after confirming that O_2_Hb and rSO_2_ had stopped decreasing after three minutes. Both days of the measurements were performed the day after when we confirmed that these postural changes could be performed without any problems. Data acquisition in the clinical setting was performed by only two persons, YT and NH. The subject was informed of the measurement details beforehand by the author, and written consent was obtained from him.

The NIRS time series data were smoothed with a 10-point moving average to remove noise in the high-frequency component. The mean and standard deviation were calculated every 10 seconds for each posture. MK, a specialist in NIRS data processing, provided ideas and suggested specific methods for the analysis of these data. AT, a specialist in NIRS research, also participated in the interpretation of the data.

Results of vital signs

During the first admission (Table 2), the patient’s systolic/diastolic BP decreased from 95/63 mmHg in the supine position to 71/50 mmHg in the first sitting position, and his BP was the lowest in the sitting position compared to those observed in other positions. The heart rate was 86-88 beats/min in the first and second supine positions but increased in the standing and second sitting positions, reaching a maximum of 98 beats/min, with an increased range of 12 beats/min. His SpO_2_ was lowest at 95% in the standing position.

**Table 2:** The patient's vital signs on day 9 HUP: head-up; SpO_2_: oxygen saturation

Vital signs	Supine	HUP	Sitting (after 30 seconds)	Sitting (after three minutes)	Standing (after 30 seconds)	Standing (After three minutes)	Sitting	Supine
Blood pressure (mmHg)	95/63	93/64	71/50	84/60	78/58	74/56	92/65	107/71
Heart rate (beats/min)	87	86	87	91	92	97	98	94
SpO_2_ (%)	98	98	97	96	98	95	98	97

On the second admission (Table 3), the systolic/diastolic BP was 141/77 mmHg in the supine position, which decreased to 125/77 mmHg in the sitting position and 117/74 mmHg in the standing position. His heart rate was lowest at 61 beats/min in the HUP position and highest at 69 beats/min in the standing position, with an increased range of 8 beats/min. His SpO_2_ was consistently 98% to 100% during the measurements.

**Table 3:** The patient's vital signs on day 100 HUP: head-up; SpO_2_: oxygen saturation

Vital signs	Supine	HUP	Sitting (after 30 seconds)	Sitting (after 3 minutes)	Standing (after 30 seconds)	Standing (after 3 minutes)	Sitting	Supine
Blood pressure (mmHg)	141/77	133/70	125/77	130/72	121/75	117/74	121/72	139/77
Heart rate (beats/min)	68	61	64	63	67	69	65	65
SpO_2_ (%)	98	98	99	100	99	99	99	100

Results of NIRS measurements

**Figure 2:**
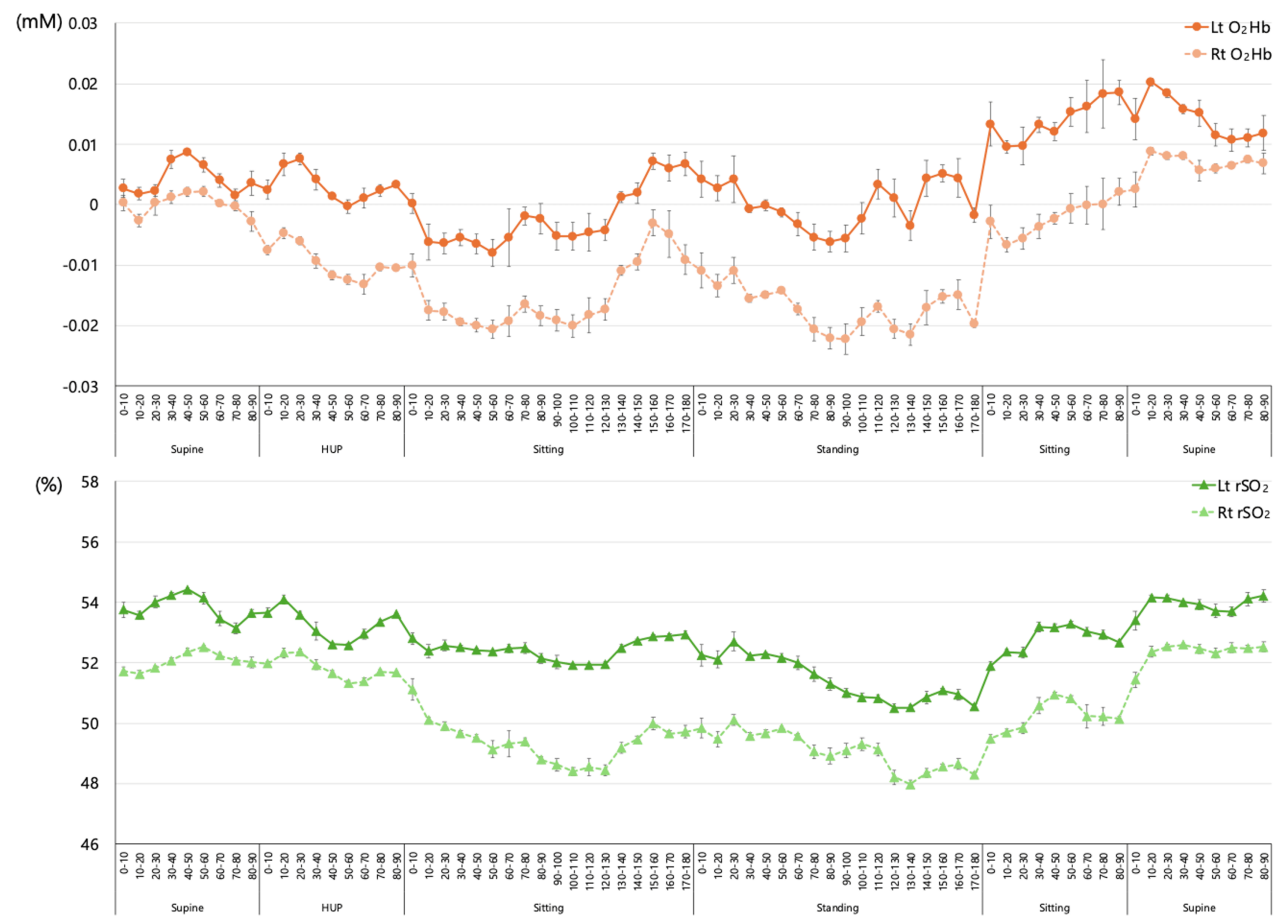
Results of the first NIRS measurement Both O_2_Hb and rSO_2_ decreased at the sitting and standing phases. The dizziness occurred approximately 10 to 20 seconds after sitting and standing, and the dizziness resolved as O_2_Hb and rSO_2_ recovered. NIRS: near-infrared spectroscopy; Lt: left; Rt: right; O_2_Hb: oxyhemoglobin; rSO_2_: regional cerebral oxygen saturation

Both O_2_Hb and rSO_2_ levels were observed to be affected in response to postural changes. At the time of first admission (Figure 2), the overall trend was a decrease in both O_2_Hb and rSO_2_ as the patient transitioned from the supine to HUP, sitting, and standing positions. These values were the lowest in the standing position. Both O_2_Hb and rSO_2_ values were lower on the right than on the left, and the left-right difference tended to be larger in the sitting and standing positions and smaller in the supine position. The rSO_2_ was approximately 52% to 53% in the first reference supine position, with a minimum value of 47.6% in the standing position. The dizziness occurred approximately 10 to 20 seconds after sitting and standing, and the dizziness resolved as O_2_Hb and rSO_2_ recovered.

**Figure 3:**
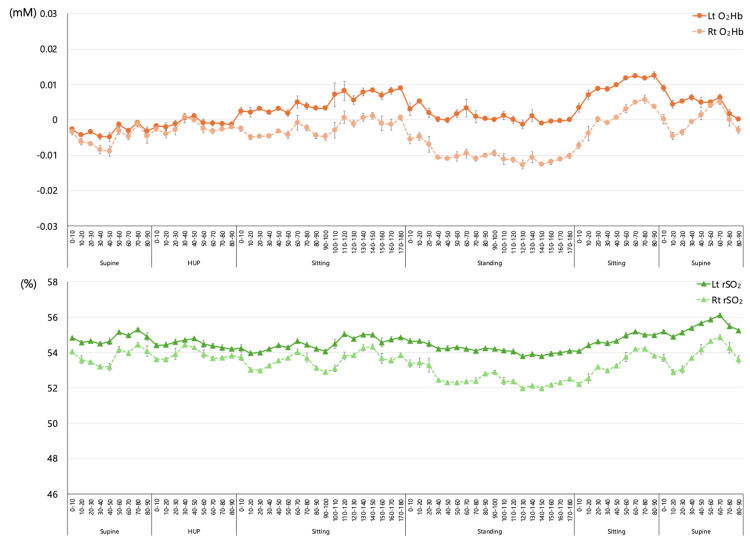
Results of the second NIRS measurement Both O_2_Hb and rSO_2_ decreased in the standing position, although the changes in O_2_Hb and rSO_2_ were smaller than on initial admission and were maintained throughout bedside mobilization. No dizziness occurred during the measurement. NIRS: near-infrared spectroscopy; Lt: left; Rt: right; O_2_Hb: oxyhemoglobin; rSO_2_: regional cerebral oxygen saturation

During the second admission (Figure 3), both the O_2_Hb and rSO_2_ levels decreased only in the standing position. Overall, the changes in O_2_Hb and rSO_2_ during the transition to the upright position were milder than in the first measurement. The left-right differences in O_2_Hb and rSO_2_ tended to be larger in the sitting and standing positions but were lower than those observed during the first hospitalization. The rSO_2_ was approximately 54% to 55% in the first reference supine position, with a minimum value of 51.8% in the standing position. However, no dizziness occurred during the measurement.

## Discussion

Although there was one report [12] that evaluated rSO_2_ during HUP tilt in patients with chronic HF, no studies have focused on patients with acute exacerbation of chronic HF or bedside mobilization. This highlights the potential of wearable NIRS devices for providing novel clinical insights and individualized care pathways in these underexplored contexts.

Ichijo et al. [13] reported that O_2_Hb concentrations measured by NIRS reflect regional cerebral blood flow, and Rifai et al. [14] suggested that rSO_2_ serves as a useful marker of cerebral blood perfusion in HF patients. Moreover, Kim et al. [10] demonstrated in healthy individuals that dynamic changes in total Hb correlate with OH symptoms during postural transitions. In this study, O_2_Hb and rSO_2_ levels were utilized as markers of cerebral blood flow, and their relationship with OH was analyzed.

During the first measurement, the patient met the diagnostic criteria for OH (a systolic BP drop ≥20 mmHg) in both the sitting and standing positions, whereas during the second measurement, this criterion was met only in the standing position. The greater decreases in O_2_Hb and rSO_2_ observed during the first measurement compared to the second suggest a correlation between the severity of OH and the magnitude of cerebral hemodynamic changes. However, differences in various background factors, such as baseline BP, cardiac function (e.g., LVEF), diuretic treatment progress, body weight, and medications, likely contributed to the observed variability in O_2_Hb and rSO_2_. Thomas et al. [8] previously noted that the duration of hypotension and cerebral hypoperfusion, rather than the absolute magnitude of BP decline, determines the occurrence of presyncope. Although the magnitude of the SBP drop was similar between the two measurements, the persistence of low baseline BP and cerebral hypoperfusion during the first measurement may explain the dizziness observed in this case.

Previous studies have highlighted the delayed recovery of cerebral oxygenation dynamics in individuals receiving antihypertensive treatment [15]. Furthermore, OH encompasses several variants, including initial OH, delayed BP recovery and sustained OH [6]. In this case, the observed prolonged decrease in cerebral hemodynamics and oxygenation dynamics, particularly after the first 10 seconds, suggests a pattern consistent with delayed BP recovery or sustained OH. This aligns with findings by Mol et al. [7], which emphasize that cerebral autoregulation may not immediately compensate for BP decreases after standing up, even in healthy individuals. Specifically, their study noted that differences in O_2_Hb responses between sitting and standing positions could not be explained solely by BP changes but were instead dependent on the efficiency of cerebral autoregulation during postural transitions. In this case, both O_2_Hb and rSO_2_ were found to decrease further in the standing position compared to the sitting position and remained low over time during both measurements. These findings suggest that impaired cerebral autoregulation may have exacerbated cerebral hypoperfusion, which, in combination with the observed postural BP changes, likely contributed to the clinical symptoms observed in this patient.

Reported rSO_2_ values in healthy adults range from 60% to 70% [16]. Rifai et al. [14] found that the mean rSO_2_ in stable chronic HF patients was 67.4% in the sitting position. Kharraziha et al. [12] reported a decrease in rSO_2_ from 67.0% in the supine posture to 63.9% at a 70° head-up tilt in HF patients, a 3.1% reduction. Fraser et al. [17] noted that a decline in rSO_2_ in HF patients correlates with reduced cerebral blood flow during upright posture. In this case, rSO_2_ remained within the 50% range throughout the study, with the lowest value observed during the standing posture. This finding aligns with previous reports, suggesting impaired cerebral blood flow due to postural changes. Interestingly, Imai et al. [18] documented rSO_2_ values of 50% to 60% in the sitting position among patients with acute HF, with no values below 50%. In contrast, this patient's rSO_2_ dropped to an exceptionally low 47.6% during the first measurement, potentially reflecting factors such as OH, decreased cardiac output or impaired cerebral autoregulation contributing to reduced cerebral blood flow.

However, this study has limitations, including its focus on a single patient, its inability to accurately quantify subjective symptoms, and the lack of continuous BP monitoring. Consequently, a definitive causal link between clinical observations and cerebral hemodynamics cannot be established. Future studies with larger sample sizes and more comprehensive data collection are warranted to validate these findings and expand upon their clinical implications.

## Conclusions

This report highlights the potential association between fluctuations in O_2_Hb, rSO_2_, and OH during mobilization in patients with acute exacerbation of chronic HF. Additionally, it underscores the potential of wearable NIRS as a non-traditional method for monitoring cerebral circulatory status in heart failure patients, aiding in risk management and the assessment of OH status. However, given the inherent limitations of a single-case study, further investigations involving larger cohorts are necessary to validate these findings and assess their broader clinical applicability.
